# Obeticholic Acid: A New Era in the Treatment of Nonalcoholic Fatty Liver Disease

**DOI:** 10.3390/ph11040104

**Published:** 2018-10-11

**Authors:** Ludovico Abenavoli, Tetyana Falalyeyeva, Luigi Boccuto, Olena Tsyryuk, Nazarii Kobyliak

**Affiliations:** 1 Department of Health Sciences, University “Magna Graecia”, Viale Europa-Germaneto, 8810 Catanzaro, Italy; 2 School of Medicine, Taras Shevchenko National University of Kyiv, Volodymyrska Street 64/13, 01601 Kiev, Ukraine; tfalalyeyeva@gmail.com (T.F.); tsyryuk@mail.ru (O.T.); 3 Greenwood Genetic Center, Greenwood, SC 29646, USA; lboccuto@ggc.org; 4 School of Health Research, Clemson University, Clemson, SC 29646, USA; 5 Department of Endocrinology, Bogomolets National Medical University, Pushkinska 22a, 01610 Kiev, Ukraine; nazariikobyliak@gmail.com

**Keywords:** nonalcoholic fatty liver disease, nonalcoholic steatohepatitis, fibrosis, obeticholic acid, farnesoid X receptor, metabolism

## Abstract

The main treatments for patients with nonalcoholic fatty liver disease (NAFLD) are currently based on lifestyle changes, including ponderal decrease and dietary management. However, a subgroup of patients with nonalcoholic steatohepatitis (NASH), who are unable to modify their lifestyle successfully, may benefit from pharmaceutical support. Several drugs targeting pathogenic mechanisms of NAFLD have been evaluated in clinical trials for the treatment of NASH. Farnesoid X receptor (FXR) is a nuclear key regulator controlling several processes of the hepatic metabolism. NAFLD has been proven to be associated with abnormal FXR activity. Obeticholic acid (OCA) is a first-in-class selective FXR agonist with anticholestatic and hepato-protective properties. Currently, OCA is registered for the treatment of primary biliary cholangitis. However, promising effects of OCA on NASH and its metabolic features have been reported in several studies.

## 1. Introduction

Nonalcoholic fatty liver disease (NAFLD) is defined as the presence of fat in the liver (hepatic steatosis) either on imaging or on liver histology, and after the exclusion of secondary causes of fat accumulation in the liver (e.g., significant alcohol consumption, certain medications, and other medical conditions) [1,2]. The histopathological presentation of NAFLD covers a fairly large spectrum, including hepatic steatosis and nonalcoholic steatohepatitis (NASH), defined by liver steatosis associated with inflammation and ballooning of the liver cells, with or without fibrosis [3]. In addition to the disorders strictly related to the liver, patients with NAFLD present with an increased frequency of metabolic conditions including obesity, type 2 diabetes, chronic kidney disease, hypertension, and colorectal malignant neoplasm [4,5]. Lifestyle is an important factor in the development and progression of NAFLD [6]. High fructose intake combined with a sedentary lifestyle is associated with higher incidence rates, especially for NASH [7]. NASH is closely linked to metabolic syndrome, defined by the occurrence of at least five of the following features: Abdominal obesity, hypertriglyceridemia, low high-density lipoprotein levels, hypertension, and elevated fasting plasma glucose [8]. Moreover, in light of the latest long-term studies, a bi-directional mutual relationship between NASH and various components of metabolic syndrome exists [9].

Currently, several pathogenic hypotheses on NAFLD development have been discussed. Both the “two-hit”, and the more recent “multi parallel hit” hypotheses suggest that many factors, such as diet, insulin resistance, hormones secreted by adipose tissue, gut microbiota, and genetics, influence fat accumulation in the hepatocytes, and expose the liver to oxidative stress, inflammation unbalance, cellular necrosis, and fibrosis [10,11].

The management of NAFLD should consist of treating liver disease as well as the associated metabolic comorbidities such as obesity, hyperlipidemia, insulin resistance, and type 2 diabetes [12]. Given that patients with NAFLD without steatosis or any fibrosis have excellent prognosis from a liver standpoint, pharmacological treatments aimed primarily at improving liver disease should generally be limited to those with biopsy-proven NASH and fibrosis [12]. Therapy is mainly based on lifestyle changes to decrease body weight, such as healthy diet and physical activity [13], and in some cases the use of metformin, thiazolidinediones, lipid-lowering drugs, and anti-tumor necrosis factor (TNF)-α agents, is required to treat the associated metabolic conditions [12,14]. Alternatively, recent studies have suggested that use of omega-3 fatty acids can decrease hepatic fat content [15,16,17], while antioxidant [6,18] and probiotic [19,20,21,22] supplementation may attenuate liver damage.

Significant lifestyle modifications are not easy to achieve or maintain on a long-term basis for some individuals. In addition, some patients with advanced liver disease may require personalized treatment [23]. Until now, no pharmaceutical protocol has been approved specifically for NAFLD and its progressive form NASH [24]. Therefore, some controlled trials targeting these disorders are currently ongoing. These trials are registered in open databases such as ClinicalTrials.gov (https://clinicaltrials.gov). The therapeutic targets of this new generation of drugs include some pathogenetic steps recently identified in the pathogenesis and the progression of this disease. In particular, these factors are insulin resistance, adi pokines/cytokines synthesis, oxidative stress, dysregulation of both lipid and carbohydrate metabolism, and fibrogenesis development [13,24]. Most of the reported factors are regulated by the farnesoid X receptor (FXR), a nuclear hormone receptor regulated by bile acids (BAs). Therefore, positively targeting FXR may represent a successful strategy for the pharmacological treatment of NASH [25,26].

## 2. Obeticholic Acid: Mechanism of Action

Obeticholic acid (OCA) is a first-in-class selective FXR agonist with anticholestatic and hepato-protective properties [27]. OCA is a 6a-ethyl selective derivative from the primary human bile acid, chenodeoxycholic acid. The chemical modification stimulates FXR activity approximately 100-fold more intensely than chenodeoxycholic acid, the natural FXR agonist in humans, and shows high selectivity with minimal activity to the other BAs receptor, G protein-coupled bile acid receptor 1 (GPBAR1 or TGR5) [28].

The FXR nuclear receptor is expressed in the liver, intestines, adrenal glands and kidneys. This nuclear receptor is significantly involved in the synthesis and enterohepatic circulation of BAs [29]. FXR is a central factor in transcriptional regulation of the hepatocellular bile formation network, that represses (via interaction with HNF4 in rats and GR in humans) hepatic BAs uptake (NTCP) and (via SHP) synthesis (CYP7A1 and CYP8B1), promotes bile secretion via induction of canalicular transporters (BSEP, MRP2, ABCG5/8, MDR3) and induces BAs elimination via alternative export systems at the hepatic basolateral (sinusoidal) membrane (OSTα/β) [30]. Intestinal bile salt activated FXR induces the expression of fibroblast growth factor 19 (FGF19, and its mouse homolog Fgf15), which is secreted into portal blood to down-regulate hepatic BAs synthesis via fibroblast growth factor receptor 4 (FGFR4) and B-Klotho signaling in the liver [31,32]. Activation of FXR in the ileum also inhibits the uptake of BAs by downregulating the sodium-dependent bile acid transporter [29,33,34,35]. In summary, OCA as an FXR agonist is a master regulator of cholesterol lipoprotein and BAs metabolism, it modulates immuno-inflammatory, and fibrogenic responses [29].

## 3. Obeticholic Acid: Animal Data

OCA has been repeatedly proven to enhance insulin sensitivity, control glucose homeostasis, modulate lipid metabolism, and exert anti-inflammatory and anti-fibrotic effects in hepatic, renal, and intestinal tissues, where FXR is mainly expressed [36].

OCA treatment of Zucker fa/fa rats, with at the dosage of 10 mg/kg, has been shown to generate a protective effect against ponderal increase and fat accumulation in hepatic and muscular tissues. Studies have also shown how OCA treatment has reversed insulin resistance assessed by insulin responsive substrate-1 phosphorylation on serine 312 in liver and muscles. FXR treatment has been shown to lower free fatty acid synthesis in muscles and liver [37], and to reduce the expression in liver of genes involved in anabolic pathways, such as lipogenesis and gluconeogenesis [37]. Similar effects on lipid metabolism have been demonstrated on apolipoprotein E-deficient (ApoE-/-) mice. Treatment with OCA at 10 mg kg^–1^ day^–1^ for 12 weeks decreases the hepatic expression of the sterol regulatory element binding protein 1c (SREBP-1c), resulting in lower triglyceride and cholesterol content in the liver and amelioration of hyperlipidemia [38]. Bile deficiency, due to a liver specific disruption of cytochrome P450 reductase (Hrn), results in the development of hepatic steatosis in murine models. Treatment with 0.025% OCA for 3 weeks ameliorates hepatic steatosis and decreases liver triglycerides [39]. On the other hand, a recent study investigated OCA effects in murine models, with wild type mice showing significant weight gain following an atherogenic diet, while appetite-dysregulated *Alms1* mutant foz/foz mice, fed the same diet, eventually showed metabolic obesity and diabetes. OCA administration resulted in improvement of adipose indices, glucose tolerance, and steatosis in the presence of milder metabolic disorders while it failed to improve these factors in mice with morbid obesity and diabetes [40].

OCA also exerts significant anti-inflammatory and anti-fibrotic activities, selectively inhibiting the processes of liver inflammation mediated by the nuclear factor (NF)-κB while maintaining or enhancing the cell survival response [41]. Other metabolic properties of OCA have been investigated, such as the impact of high-fat diet (HFD) on arachidonic acid metabolism in the liver and the role of OCA in eicosanoid biosynthetic pathways and NF-κB signaling. An imbalance in arachidonate metabolism was described in association with NAFLD, but FXR activation reprogrammed it by inducing P450 epoxygenase expression and epoxyeicosatrienoic acids (EET) production. In vitro FXR-mediated NF-κB inhibition requires active P450 epoxygenases [42]. In addition, OCA is capable of inhibiting vascular smooth muscle cell inflammation via the downregulation of NF-κB-dependent expression of inducible nitric oxide synthase (iNOS) and cyclooxygenase-2 (COX-2) [43].

Recent studies on two mouse models of obesity, a diet-induced model (DIO-NASH) and Lep ob/ob model (ob/ob-NASH), compared the efficacy of three novel agents, liraglutide (0.2 mg/kg, SC, BID), obeticholic acid (OCA, 30 mg/kg PO, QD), or elafibranor (30 mg/kg PO, QD) for eight weeks, on NASH histological features resolution as assessed by the nonalcoholic fatty liver disease activity score (NAS) and fibrosis staging system. Liraglutide is a long-acting GLP-1 analogue and has been shown to cause dose-dependent weight loss, decrease glycosylated hemoglobin (HbA1c), and improve β-cell function in overweight patients with type 2 diabetes. In animal models, liraglutide have been shown to improve liver enzymes, oxidative stress, and hepatic steatosis [44]. Elafibranor is an agonist of the peroxisome proliferator–activated receptor-α and peroxisome proliferator–activated receptor-δ [45]. Elafibranor improves insulin sensitivity, glucose homeostasis, lipid metabolism, and reduces inflammation [45]. Liraglutide, OCA, and elafibranor have proved to be able to produce beneficial effects in murine DIO-NASH and ob/ob-NASH models. When reduction of body weight was assessed in the same models, liraglutide and elafibranor, but not OCA, were successful. Liraglutide also improved steatosis scores in DIO-NASH mice only. Histopathological scores of hepatic steatosis and inflammation improved in both models after treatment with elafibranor and OCA, but only elafibranor use resulted in a decrease in fibrosis severity [46].

The main histopathological trait in NASH is hepatic fibrosis, which is dependent on the activation of hepatic stellate cells (HSCs) [36]. FXR is expressed in HSCs and regulates their activation, playing a pivotal role in the fibrogenic process [47]. In line with this concept, it has been demonstrated in several models that OCA can protect against liver fibrosis by inhibiting HSC activation [48]. In the thioacetamide-induced hepatic fibrosis in rats, for example, OCA prevents fibrosis progression, reverses fibrosis and cirrhosis development, and noticeably reduces portal hypertension [36,49]. A recent study investigated the impact of OCA on the development of NASH employing melanocortin 4 receptor-deficient (MC4R-KO) mice [50]. OCA administration effectively prevented chronic inflammation and liver fibrosis.

Hepatic crown-like structure (hCLS) is a distinguishing histological structure typical of NASH, which triggers death-induced interstitial fibrosis in liver cells. Treatment with OCA has been shown to significantly limit hCLS formation even after MC4R-KO mice developed NASH, thereby inhibiting the progression of liver fibrosis. The OCA effect was mediated by the suppression of metabolic stress-induced p53 activation and cell death in hepatocytes [50].

Thus, multiple FXR-mediated activities of OCA have been proven pre-clinically, such as increased insulin sensitivity, reduced lipid synthesis and fat accumulation, hepatocyte protection against bile acid-induced cytotoxicity, anti-inflammatory effects in liver, and prevention and reversal of liver fibrosis. Such properties suggest a promising role for this compound as a novel therapeutic agent for NAFLD and NASH.

## 4. Obeticholic Acid: Clinical Trials

Two major Phase II studies have evaluated the safety and efficacy of OCA in patients with biopsy proven NASH [51] and NAFLD with type 2 diabetes [52], respectively. Findings from clinical trials are summarized in Table 1.

Considering the preclinical findings supporting the therapeutic potential of FXR agonists in the regulation of glucose and lipid metabolism, a phase IIa study was designed to test OCA in patients with type 2 diabetes and NAFLD [52]. This was a multicenter, placebo (n = 23) controlled study that evaluated two oral daily doses of OCA: 25 mg (n = 20) and 50 mg (n = 21) for six weeks [52]. The primary endpoint in the study was the change, post- versus pre-treatment, in the glucose infusion rate (GIR) during the low and high dose insulin infusion periods. This endpoint was met, with statistically significant increases in GIR at 25 mg, as well as for both OCA doses combined, at both insulin infusion levels [52]. Overall, the administration of OCA was well tolerated and generated an increase in insulin sensitivity and a reduction of markers of liver inflammation and fibrosis. Noticeably, OCA promoted a prominent dose-related increase in the serum levels of FGF19, which confirmed FXR activation and suggests a mechanism of action for the OCA-induced improvement in insulin sensitivity and weight loss [36,52].

In the phase IIb of the FLINT trial, 283 subjects with histologically confirmed NASH were treated with OCA for 72 weeks. The primary outcome, a decrease in the NAFLD fibrosis score (NAS) by at least 2 points without worsening of fibrosis from baseline to the end of treatment, was achieved in 45% in the OCA group vs. 21% in the placebo group (*p* = 0.0002). Improvement in fibrosis was observed in 35% in the OCA group and 19% in the placebo group. OCA also was associated with improvement in steatosis, lobular inflammation, and hepatocyte ballooning but not with NASH resolution [51]. A phase 2 randomized, double-blind, placebo-controlled trials in Japan (FLINT-J trial) showed that high doses of OCA (40 mg/day) significantly resolved NASH compared with a placebo (38 vs. 20%, *p* = 0.049). The improvement of fibrosis registered in the cases exposed to OCA was similar to that in the placebo group [51]. The discrepancy between the FLINT and the FLINT-J study may be due to the fact that in the FLINT-J study, NASH with mild fibrosis at entry is prevalent. Some patients in the OCA group refused post-treatment liver biopsy, and those were classified as non-responders. OCA proved to be more efficient than a placebo in ameliorating serum alanine aminotransferase (ALT) levels and hepatocyte histology. OCA therapy also lowered body weight, which by itself can improve histology. Therefore, it has been questioned if the effects of OCA depend on ponderal changes. Hameed et al. reported a post hoc analysis of the FLINT trial to address this issue [53]. As expected, the NAS improved more in those with weight decrease in both the OCA (−2.4 vs. −1.2, *p* < 0.001) and placebo-treated patients (−1.2 vs. −0.5, *p* = 0.03); Similar trends were observed for ALT levels, although the difference did not reach statistical significance. Hence, it has been inferred that the effects of OCA on histology is independent and additive to weight loss [47]. However, favorable effects of ponderal decrease on alkaline phosphatase, lipids, and blood glucose seen in placebo-treated patients were absent or reversed in the OCA treatment. These findings highlight the value of evaluating the metabolic impact of novel treatment approaches of NASH [53]. An international, phase 3 study, REGENERATE (ClinicalTrials.gov: NCT02548351) in patients with non-cirrhotic NASH with liver fibrosis and REVERSE (ClinicalTrials.gov: NCT03439254) for patients with compensated cirrhosis due to NASH, are now ongoing. If similar results are confirmed, unravelling the molecular mechanisms and/or selective modulation of FXR activity might lead the way towards an ideal pharmacotherapy for NASH.

## 5. Obeticholic Acid: Safety and Tolerability

Few side effects have been reported in association with OCA. Those that have been reported were commonly mild to moderate, as a pruritus, dyslipidemia, fatigue, headache, and gastrointestinal side effects. Pruritus is the most common adverse effect in the treated cohorts requiring dose adjustment and/or discontinuation of the administration. The current evidence suggests that there is an increased frequency and severity of pruritus, especially in OCA treatment groups at higher doses. In the randomized FLINT clinical trial 23% of 141 patients in the OCA group developed pruritus compared with 6% in the placebo group [51]. However, this symptom can be managed in most patients by the use of bile acid sequestrants, antihistamines, dose reduction, or symptomatic treatment [29].

Treatment with OCA has been associated with an increase in LDL-cholesterol and a decrease in HDL-cholesterol and triglycerides. Recently, to highlight this side effect and to try to find ways for its correction, a trial on the association between OCA and statins began (ClinicalTrials.gov: NCT02633956). In this study both liver-related outcomes as well as dyslipidemia are being investigated. This Phase 2, double-blind, randomized, placebo-controlled, multicenter study, with an open-label long-term safety extension, is evaluating the effect of OCA and the subsequent addition of statin therapy on lipoprotein metabolism in subjects with NASH and fibrosis stage 1 to 4, but no evidence of hepatic decompensation. Recruitment has now been completed, and the results will be published in the near future.

## 6. Conclusions

NAFLD is a multifaceted systemic disease, with a relevant epidemiological impact. Currently, lifestyle changes with dietary restrictions and physical activity remain the only recommended treatment for NAFLD, in all patients with increased body weight [1]. Presently, OCA is registered for the treatment of primary biliary cholangitis. However, FXR is a promising molecular target for NAFLD therapy. OCA has several drawbacks, such as elevated LDL levels, itching, and high cost. However, clinical interest in potential off-label use of OCA is likely to be high, given the lack of available treatments with liver-specific effects. We can conclude that, on the basis of this literature review, OCA appears to be a novel agent with promising potential that could be considered for future treatment protocols of NASH.

## Figures and Tables

**Table 1:** Randomized clinical trials using obeticholic acid (OCA) in patients with nonalcoholic fatty liver disease (NAFLD) and nonalcoholic steatohepatitis (NASH).

Authors	Phase/Status	Patient Population	Duration	Primary/Secondary Endpoints	Intervention Dosage/Subjects	Findings
FLINT study [51] (NCT01265498)	III/Completed	Biopsy proven NASH	72 weeks	Histological Improvement in NAS (no worsening in fibrosis; and decrease in NAFLD Activity Score (NAS) of at least 2 points	25 mg OCA (n = 141) Placebo (n = 142)	Primary endpoint was achieved in 45% of the patients receiving OCA and 21% of those receiving placebo
Mudaliar et al. [52] (NCT00501592)	II/Completed	Type 2 diabetes patient with presumed NAFLD	6 weeks	Assessing changes in insulin resistance and glucose homeostasis/Hepatocellular function	25 mg OCA (n = 20) 50 mg OCA (n = 21) Placebo (n = 23)	Administration of 25 or 50 mg OCA increased insulin sensitivity, and reduced markers of liver inflammation and fibrosis
REVERSE study (NCT03439254)	III/Recruitment	Patient with compensated cirrhosis due to NASH	12 months	% subjects with improvement in fibrosis by at least 1 stage with no worsening of NASH, using NASH CRN/ % subjects with improvement in fibrosis by at least 2 stage or with NASH resolution	10 mg OCA 10 up to 25 mg OCA Placebo Totally (n = 540)	
RE-GENERATE study (NCT02548351)	III/Recruitment	Patient with non-cirrhotic NASH with liver fibrosis	18 months	% patients that achieve at least one stage of liver fibrosis improvement with no worsening of NASH, or NASH resolution with no worsening of liver fibrosis/liver-related clinical outcomes	10 mg OCA 25 mg OCA Placebo Totally (n = 2370)	
CONTROL study (NCT02633956)	II/Recruitment completed	Biopsy proven NASH with fibrosis stage 1–4	16 weeks	Effect on LDL and LDL particle concentration, LDL particle size	5 mg OCA/20 mg Atorvastatin (n = 20) 10 mg OCA/20 mg Atorvastatin (n = 21) 25 mg OCA/20 mg Atorvastatin (n = 22) Placebo/20 mg Atorvastatin (n = 21)	
